# Supplementary material for: Effect on catch efficiency and bycatch by introducing an Excluder device in the trawl fishery for lesser sandeel (Ammodytes marinus)
Source: PLoS One. 2024 Jul 31;19(7):e0306744. doi: 10.1371/journal.pone.0306744 (PMC11290672; doi:10.1371/journal.pone.0306744)
Supplement: S1 File — The raw data underlying the analysis consist of count data for number of individuals of all species caught with respectively the Excluder codend and the Grid codend, for each size class (length). The count numbers are provided for each sample of each haul. (ZIP) [file pone.0306744.s001.zip › S1 File. Meta data.pdf]

HVL = Whiting (*Merlangius merlangus*),

KNH = Grey Gurnard (*Eutrigla gurnardus*)

KUL = Haddock (*Melanogrammus aeglefinus*)

MAK = Mackerel (*Scomber scombrus*)

MJS = Saithe (*Pollachius virens*)

SIL = Herring (*Clupea harengus*)

TMB = lesser sandeel (*Ammodytes marinus*)

TOR = Cod (*Gadus morhua*)

S1, S2, ………… S23 = Haul1, Haul2, ………. Haul23

Test1 = Excluder trawl

Test2 = Standard trawl

LENGTH = Length in centimeters

SAMPLING = Fraction of total haul weight measured
