# Supplementary material for: Effect on catch efficiency and bycatch by introducing an Excluder device in the trawl fishery for lesser sandeel (Ammodytes marinus)
Source: PLoS One. 2024 Jul 31;19(7):e0306744. doi: 10.1371/journal.pone.0306744 (PMC11290672; doi:10.1371/journal.pone.0306744)
Supplement: Attachment — Submitted filename: Response to reviewers.docx [file pone.0306744.s002.docx]

**[PONE-D-24-11630] Revision required**

**Comments from Academic editor:**

| *Journal Requirements:* | |
| --- | --- |
| ***Specific comments*** | ***Author response*** |
| 1. Please ensure that your manuscript meets PLOS ONE's style requirements, including those for file naming. The PLOS ONE style templates can be found at  <https://journals.plos.org/plosone/s/file?id=wjVg/PLOSOne_formatting_sample_main_body.pdf> and  <https://journals.plos.org/plosone/s/file?id=ba62/PLOSOne_formatting_sample_title_authors_affiliations.pdf> | The manuscript has been revised to meet PLOS ONE's style requirements |
| 2. To comply with PLOS ONE submissions requirements, in your Methods section, please provide additional information regarding the experiments involving animals and ensure you have included details on (a) methods of sacrifice, (b) methods of anesthesia and/or analgesia, and (c) efforts to alleviate suffering. | The requested information has been added in the Methods section |
| 3. Thank you for stating the following in the Acknowledgments Section of your manuscript:  This study was financed by the Ministry of Food, Agriculture and Fisheries of Denmark through the GUDP (Green Development and Demonstration Program) in the project ‘MiniMakS’ (34009-20-1674). We also wish to thank the crews and teams of “S205 Ceton”, the Flume tank in Hirtshals, and Jonathan Stounberg for their cooperation and assistance in the project, and not least Greenline Fishing Gear (<http://www.greenlinefishinggear.com>) and Tor-Mo Trawl ApS (<http://www.tormotrawl.dk/>) for developing and producing the tested Excluder.  We note that you have provided funding information that is not currently declared in your Funding Statement. However, funding information should not appear in the Acknowledgments section or other areas of your manuscript. We will only publish funding information present in the Funding Statement section of the online submission form.  Please remove any funding-related text from the manuscript and let us know how you would like to update your Funding Statement. Currently, your Funding Statement reads as follows:  We wish to thank the Ministry of Food, Agriculture, and Fisheries of Denmark (<https://fvm.dk/>) for funding the work presented here through the GUDP (Green Development and Demonstration Program) in the project ‘MiniMakS’ (34009-20-1674). The funder did not play any role in the study design, data collection and analysis, decision to publish, or preparation of the manuscript.  Please include your amended statements within your cover letter; we will change the online submission form on your behalf. | We have deleted the funding information from the manuscript. This also means that the current information and wording in our Funding Statement is correct. |
| 4. Please include your full ethics statement in the ‘Methods’ section of your manuscript file. In your statement, please include the full name of the IRB or ethics committee who approved or waived your study, as well as whether or not you obtained informed written or verbal consent. If consent was waived for your study, please include this information in your statement as well. | The requested information has been added in the Methods section. |
| 5. We note that Figure 1 in your submission contains map images which may be copyrighted. All PLOS content is published under the Creative Commons Attribution License (CC BY 4.0), which means that the manuscript, images, and Supporting Information files will be freely available online, and any third party is permitted to access, download, copy, distribute, and use these materials in any way, even commercially, with proper attribution. For these reasons, we cannot publish previously copyrighted maps or satellite images created using proprietary data, such as Google software (Google Maps, Street View, and Earth). For more information, see our copyright guidelines: <http://journals.plos.org/plosone/s/licenses-and-copyright>.   We require you to either present written permission from the copyright holder to publish these figures specifically under the CC BY 4.0 license, or remove the figures from your submission:   a. You may seek permission from the original copyright holder of Figure 1 to publish the content specifically under the CC BY 4.0 license.     We recommend that you contact the original copyright holder with the Content Permission Form (<http://journals.plos.org/plosone/s/file?id=7c09/content-permission-form.pdf>) and the following text: “I request permission for the open-access journal PLOS ONE to publish XXX under the Creative Commons Attribution License (CCAL) CC BY 4.0 (<http://creativecommons.org/licenses/by/4.0/>). Please be aware that this license allows unrestricted use and distribution, even commercially, by third parties. Please reply and provide explicit written permission to publish XXX under a CC BY license and complete the attached form.”   Please upload the completed Content Permission Form or other proof of granted permissions as an "Other" file with your submission.   In the figure caption of the copyrighted figure, please include the following text: “Reprinted from [ref] under a CC BY license, with permission from [name of publisher], original copyright [original copyright year].”   b. If you are unable to obtain permission from the original copyright holder to publish these figures under the CC BY 4.0 license or if the copyright holder’s requirements are incompatible with the CC BY 4.0 license, please either i) remove the figure or ii) supply a replacement figure that complies with the CC BY 4.0 license. Please check copyright information on all replacement figures and update the figure caption with source information. If applicable, please specify in the figure caption text when a figure is similar but not identical to the original image and is therefore for illustrative purposes only. The following resources for replacing copyrighted map figures may be helpful:   USGS National Map Viewer (public domain): <http://viewer.nationalmap.gov/viewer/> The Gateway to Astronaut Photography of Earth (public domain): <http://eol.jsc.nasa.gov/sseop/clickmap/> Maps at the CIA (public domain): <https://www.cia.gov/library/publications/the-world-factbook/index.html> and <https://www.cia.gov/library/publications/cia-maps-publications/index.html> NASA Earth Observatory (public domain): <http://earthobservatory.nasa.gov/> Landsat: <http://landsat.visibleearth.nasa.gov/> USGS EROS (Earth Resources Observatory and Science (EROS) Center) (public domain): [http://eros.usgs.gov/#](http://eros.usgs.gov/) Natural Earth (public domain): <http://www.naturalearthdata.com/> | Figure 1 in the manuscript is **not** a [map/satellite] image, but has been produced by the authors in ArcGIS, based on UTM position data from the experimental vessel.  Consequently, there is no issue with copyright. |
| 6. Please review your reference list to ensure that it is complete and correct. If you have cited papers that have been retracted, please include the rationale for doing so in the manuscript text or remove these references and replace them with relevant current references. Any changes to the reference list should be mentioned in the rebuttal letter that accompanies your revised manuscript. If you need to cite a retracted article, indicate the article’s retracted status in the References list and also include a citation and full reference for the retraction notice. | The reference list has been reviewed. No changes were made. |

**Review Comments to the Author**

| *Reviewer #1: The use of soft BRD Excluder significantly reduced the bycatch ratio above certain sizes while did not affect the catch efficiency for lesser sandeel. Although it is not mandatory to be used in the local sandeel trawl fishery, its soft-netting, catching effectiveness and easy-operation properties indicated its potential to be used in other commercial fisheries and to be an alternative for rigid BRD. The results were significant for research and fishing practice, and the paper was well written and data analysis was reasonable. It is recommended to be accepted with minor revisions below:* | |
| --- | --- |
| ***Specific comments*** | ***Author response*** |
| Table 1. hauling positions (latitude and longitude data) could be removed since they are already presented in Figure 1 | Positions removed from Table 1 |
| Too many tables in the paper. Could Table 2 and 3 be transferred to figures? It is easy to see the difference between control and Excluder in a figure. | We have carefully considered this suggestion from the reviewer. However, the results presented in Table 2 and Table 3 are not available from any of the figures shown in the manuscript. Therefore, as these results are important and central for the paper we prefer keeping the existing Table 2 and 3 in the manuscript, also considering that the total number of Tables is only 6. |
| I want to see a discussion section about the mesh block of the Excluder. A larger volume of sandeel and some bycatch species need to bypass the device (in a short time?). Are the mesh blocked by the passed fishes and therefore affect the effectiveness of the device although the towing speed was not so high? | A discussion section has been added as suggested. |
|  |  |
| *Reviewer #2: The manuscript evaluates the effect of an Excluder device on catch efficiency and bycatch reduction in the trawl fishery for lesser sandeel. The study involves experimental sea trials comparing standard trawl configuration with and without the Excluder. Results indicate that the Excluder significantly reduces bycatch of non-target species such as haddock, mackerel, and grey gurnard without substantially affecting the catch efficiency of the target species.* *The study addresses a relevant issue in fisheries management by investigating the potential of the Excluder device to reduce bycatch in a targeted sandeel fishery. The manuscript is well-structured, and the methods and analyses are generally sound. However, there are a few minor corrections needed to improve clarity and accuracy.* | |
| ***Specific comments*** | ***Author response*** |
| Line 72: The scientific name “Ammodytes marinus” should be abbreviated to “A. marinus” in its second occurrence within the parentheses. | The manuscript has been revised according to the comment. |
| Line 80: Please add the scientific name for Norway pout, which is “Trisopterus esmarkii.” | The manuscript has been revised according to the comment. |
| Experimental objectives This innovative design is particularly beneficial for net drum users, as it ensures the Excluder can be easily reeled onto net drums, enhancing both operational efficiency and safety. | The manuscript has been revised according to the comment. |
| Data collection: In summary, the data collection method described has several critical flaws that need to be addressed to enhance its scientific validity. Reliance on subjective skipper estimates, lack of clarity on sample collection timing, and inconsistent sampling fractions are significant issues. Clear guidelines for sub-sampling and a thorough explanation of measurement and raising techniques are necessary. Addressing these concerns will improve the accuracy and reliability of the data collected, ensuring more robust scientific outcomes. | We agree with the reviewer that we did not describe in sufficient detail how the data collection was conducted and how we have accounted for the uncertainty due to the sampling procedure and the variation in sampling fractions. We have now described this in more detail in the revised manuscript. |
| Line 159: In Equation 1, the variables d_n and d_w need to be defined in the text. Please provide definitions for these terms to ensure clarity. | Thanks for the comment, the reviewer is correct. We should explain better. Therefore, have added text that explains in more detail what these performances measures express. |
